# Supplementary material for: Economic freedom, inclusive growth, and financial development: A heterogeneous panel analysis of developing countries
Source: PLoS One. 2023 Jul 11;18(7):e0288346. doi: 10.1371/journal.pone.0288346 (PMC10335674; doi:10.1371/journal.pone.0288346)
Supplement: S1 File — (PDF) [file pone.0288346.s001.pdf]

S1 Table Linear dynamic panel data instrumental variables GMM estimations of economic freedom index, inclusive growth, and sub-dimensions of financial development index

	1	2	1	2	1	2	1	2	1	2	1	2
	FIDIX	FIDIX	FIAIX	FIAIX	FIEIX	FIEIX	FMDIX	FMDIX	FMAIX	FMAIX	FMEIX	FMEIX
efio	0.064	0.007	-0.015	-0.120	0.042	0.165	0.021	0.028	0.163	0.157	0.072	0.079
	(21.29)***	(3.40)***	(-3.01)**	(-15.63)***	(5.67)***	(15.59)***	(5.04)***	(5.45)***	(58.54)***	(11.53)***	(6.37)***	(7.48)***
lngdppc	0.154	0.162	0.336	0.326	0.091	0.028	0.170	0.174	-0.093	-0.062	-0.309	-0.258
	(67.40)***	(50.89)***	(226.54)***	(51.19)***	(16.61)***	(3.55)***	(69.28)***	(37.24)***	(-59.16)***	(-11.85)***	(-43.41)***	(-26.62)***
lnpopg	-0.0003	-0.003	0.017	0.014	-0.016	-0.013	-0.003	-0.001	0.006	0.005	-0.007	-0.010
	(-3.80)***	(-58.50)***	(293.19)***	(76.57)***	(-57.65)***	(-32.00)***	(-49.59)***	(-12.34)***	(225.93)***	(45.56)***	(-36.66)***	(-28.34)***
lnGCF	-0.013	-0.023	0.005	-0.004	0.017	0.039	-0.017	-0.020	0.030	0.020	0.105	0.074
	(-24.47)***	(-23.89)***	(14.48)***	(-3.13)**	(6.84)***	(10.23)***	(-14.91)***	(-10.50)***	(42.59)***	(10.97)***	(44.36)***	(24.68)***
exogshock		-0.003		0.011				0.013		-0.009		-0.013
		(-4.70)***		(5.48)***				(10.43)***		(-8.92)***		(-15.76)***
endoshock		0.354		0.566				-0.331		-0.431		-0.407
		(9.97)***		(11.88)***				(-5.55)***		(-11.46)***		(-12.78)***
constant	-1.137	-1.174	-2.738	-2.774	-0.757	-1.908	-1.076	-0.728	-0.354	0.195	0.277	1.045
	(-45.71)***	(-20.44)***	(-70.72)***	(-47.20)***	(-16.43)***	(-20.40)***	(-34.07)***	(-10.98)***	(-31.24)***	(3.91)***	(6.64)***	(19.76)***
Wald chi^2	24777.82***	45506.54***	512210.59***	284843.27***	14125.98***	6030.20***	208043.84***	29358.09***	74683.85***	9962.50***	10351.80***	14771.37***
Sargan test	68.562(1.00)	63.413(1.00)	70.866(1.00)	60.273(1.00)	67.782(1.00)	66.879(1.00)	70.749(1.00)	68.408(1.00)	70.017(1.00)	64.177(1.00)	69.894(1.00)	69.660(1.00)
Autocorrelation:												
ART (1)	1.166(0.24)	0.424(0.67)	3.820(0.00)	3.909(0.00)	-2.641(0.01)	-2.086(0.04)	-0.816(0.42)	-0.850(0.40)	-0.572(0.57)	-0.476(0.63)	-1.169(0.24)	-1.089(0.28)
ART (2)	-0.055(0.96)	-0.033(0.97)	2.298(0.02)	2.828(0.01)	-1.985(0.05)	-2.015(0.05)	0.783(0.43)	0.782(0.43)	-1.862(0.06)	-2.070(0.04)	-0.349(0.73)	-0.490(0.62)
No of Instruments	141	180	141	180	141	180	141	180	141	180	141	180
observation	648	648	648	648	648	648	648	648	648	648	648	648

Note: *** denote 1% significance level, ** denote 5% significance level. Z-statistics are in parentheses. 1 = model without exogenous and endogenous shocks, 2 = model with exogenous and endogenous shocks. ART(1) and (2) as well as Sargan tests p-values are parentheses.

S2 Table Robust check analysis of economic freedom, inclusive growth, and financial development

	PCSE -1	PCSE - 2	GLS	PCSE -1	PCSE - 2	GLS	PCSE -1	PCSE - 2	GLS
	FDIX	FDIX	FDIX	FIIX	FIIX	FIIX	FMIX	FMIX	FMIX
efio	0.043	0.033	0.040	0.062	0.038	0.056	0.023	0.028	0.004
	(4.64)***	(3.24)***	(2.26)**	(6.90)***	(4.08)***	(3.94)***	(1.26)**	(1.41)**	(0.09)**
lngdppc	0.087	0.088	0.089	0.113	0.115	0.112	0.060	0.059	0.061
	(338.20)***	(39.00)***	(16.62)***	(38.16)***	(40.57)***	(49.20)***	(24.22)***	(24.34)***	(8.83)***
lnpopg	-0.005	-0.006	-0.004	-0.015	-0.015	-0.014	0.004	0.003	0.005
	(-2.20)**	(-2.57)**	(-1.64)	(-6.44)***	(-6.70)***	(-8.49)***	(1.14)	(0.78)	(1.35)
lnGCF	0.005	0.006	0.005	0.001	-0.0003	0.002	0.009	0.012	0.008
	(15.98)***	(11.51)***	(6.83)***	(2.65)**	(-0.75)	(3.47)***	(19.82)***	(14.42)***	(6.61)***
exogshock		-0.018			0.007			-0.042	
		(-5.64)***			(2.72)**			(-7.04)***	
endoshock		-0.010			-0.050			0.032	
		(-2.15)**			(-8.67)***			(5.88)***	
constant	-0.794	-0.687	-0.795	-0.882	-0.743	-0.883	-0.688	-0.616	-0.588
	(-17.44)***	(-11.65)***	(-12.01)***	(-19.72)***	(-13.74)***	(-14.91)***	(-7.98)***	(-6.03)***	(-3.28)***
R-squared	0.410	0.411		0.546	0.555		0.400	0.400	
Wald chi^2	4074.25***	16344.27***	939.34***	2678.56***	10957.63***	6303.60***	2284.40***	3523.98***	182.23***
Autocorrelation	No	No	No	No	No	No	No	No	No
observation	648	648	648	648	648	648	648	648	648

Note: *** denote 1% significance level, ** denote 5% significance level. Z-statistics are in parentheses. 1 = model without exogenous and endogenous shocks, 2 = model with exogenous and endogenous shocks. ART(1) and (2) as well as Sargan tests p-values are in parentheses.

S3 Table Robust check analysis of economic freedom index, inclusive growth, and sub-dimensions of financial development index

	1	2	1	2	1	2	1	2	1	2	1	2
	FIDIX	FIDIX	FIAIX	FIAIX	FIEIX	FIEIX	FMDIX	FMDIX	FMAIX	FMAIX	FMEIX	FMEIX
efio	0.231	0.217	-0.098	-0.149	0.042	0.043	0.078	0.079	0.098	0.074	-0.163	-0.131
	(15.93)***	(14.27)***	(-4.86)***	(-7.46)***	(2.25)**	(2.10)**	(3.78)***	(3.43)***	(4.34)***	(3.25)***	(-6.74)***	(-4.86)***
lngdppc	0.069	0.071	0.177	0.182	0.057	0.056	0.035	0.035	0.092	0.094	0.039	0.035
	(38.41)***	(36.49)***	(38.31)***	(41.61)***	(12.18)***	(13.51)***	(12.98)***	(11.70)***	(30.36)***	(31.36)***	(9.10)***	(8.65)***
lnpopg	0.010	0.010	-0.034	-0.036	-0.016	-0.017	0.011	0.010	0.014	0.013	-0.015	-0.018
	(5.07)***	(5.00)***	(-5.37)***	(-5.55)***	(-3.81)***	(-3.78)***	(3.61)***	(3.12)**	(2.57)**	(2.38)**	(-2.35)**	(-2.81)**
lnGCF	0.001	4.12e-06	-0.0004	-0.003	0.002	0.003	0.010	0.012	0.004	0.002	0.015	0.022
	(2.09)**	(0.01)	(-0.92)	(-4.54)***	(3.79)***	(4.68)***	(3.61)***	(14.99)***	(9.26)***	(3.59)***	(19.15)***	(14.31)***
exogshock		0.011		0.015		-0.009		-0.037		0.011		-0.106
		(2.67)**		(5.51)***		(-1.66)*		(-6.62)***		(2.53)**		(-8.52)***
endoshock		-0.031		-0.103		0.006		0.022		-0.052		0.116
		(-5.57)***		(-21.47)***		(0.47)		(3.23)***		(-16.13)***		(10.62)***
constant	-1.367	-1.309	-0.831	-0.549	-0.107	-0.087	-0.685	-0.603	-1.160	-1.032	0.121	0.176
	(-23.53)***	(-21.24)***	(-8.51)***	(-5.18)***	(-1.31)	(-0.84)	(-8.32)***	(-6.04)***	(-12.09)***	(-9.41)***	(1.01)	(10.62)***
R-squared	0.593	0.500	0.538	0.554	0.494	0.400	0.407	0.513	0.500	0.500	0.535	0.500
Wald chi2	6340.58***	11633.82***	4425.89***	19095.59***	395.00***	1118.46***	1220.12***	3761.38***	1926.69***	6201.29***	750.47***	956.32***
Autocorrelation	No	No	No	No	No	No	No	No	No	No	No	No
observation	648	648	648	648	648	648	648	648	648	648	648	648

Note: *** denote 1% significance level, ** denote 5% significance level, * denote 10% significance level. 1 = model without exogenous and endogenous shocks, 2 = model with exogenous and endogenous shocks. Z-statistics are in parentheses.

S4 Table Analysis of inclusive growth and sub-dimensions of economic freedom and financial development

	1	2	1	2	1	2	1	2	1
	FDIX	FDIX	FIIX	FIIX	FMIX	FMIX	FIDIX	FIDIX	FIAIX
efipr	0.030***	0.033***	-0.014	-0.010	0.072***	0.076***	0.026**	0.029**	-0.036**
efije	0.005	0.005	0.013	0.018	-0.003	-0.008	-0.007	-0.001	0.040
efigi	0.016	0.017	0.058***	0.061***	-0.026	-0.028	0.101***	0.105***	-0.011
efitb	-0.659***	-0.674***	-0.525***	-0.559***	-0.779***	-0.774***	-0.916***	-0.956***	-0.083**
efigs	0.054***	0.055***	0.003	0.009	0.104***	0.100***	0.080***	0.088***	-0.966***
efifh	-0.006	-0.007	-0.009	-0.013	-0.004	0.0004	0.006	0.001	-0.032
efibf	0.010	0.010	0.074***	0.071***	-0.056	-0.052	0.067***	0.062**	0.041**
efilf	0.009	0.009	0.003	0.012	0.016	0.005	0.033**	0.046***	-0.039**
efimf	0.118***	0.096**	0.176***	0.135***	0.058*	0.054*	0.172***	0.125***	-0.0004
efitf	-0.019	0.005	0.115***	0.175***	-0.153***	-0.165***	0.105**	0.175***	0.251***
efiif	-0.067***	-0.067***	-0.068***	-0.072***	-0.064***	-0.060***	-0.099***	-0.103***	-0.012
efiff	0.116***	0.105***	0.094***	0.076***	0.135***	0.131***	0.151***	0.131***	0.024*
lngdppc	0.079***	0.079***	0.086***	0.087***	0.070***	0.069***	0.043***	0.045***	0.149***
R-squared	0.605	0.610	0.681	0.698	0.392	0.399	0.465	0.483	0.587
	2	1	2	1	2	1	2	1	2
	FIAIX	FIEIX	FIEIX	FMDIX	FMDIX	FMAIX	FMAIX	FMEIX	FMEIX
efipr	-0.030**	-0.031**	-0.032**	0.036***	0.039***	0.117***	0.120***	0.062***	0.067***
efije	0.045	0.001	0.001	0.018	0.015	-0.030**	-0.026**	-0.0004	-0.017**
efigi	-0.007	0.079***	0.079***	0.006	0.005	-0.052**	-0.049*	-0.022	-0.030*
efitb	-0.133***	-0.473***	-0.471***	-0.817***	-0.816***	-0.575***	-0.612***	-0.966***	-0.921***
efigs	-0.088***	0.034***	0.034***	0.126***	0.124	0.097***	0.103***	0.085***	0.069***
efifh	-0.038	0.004	0.004	-0.015	-0.013	0.017	0.012	-0.010	0.004
efibf	0.036*	0.111***	0.111***	-0.037	-0.034	0.014	0.010	-0.154***	-0.141***
efilf	-0.026*	0.018	0.018	0.018	0.111	0.026*	0.036***	-0.023	-0.059***
efimf	-0.062*	0.372***	0.375***	0.096**	0.089**	0.143***	0.099**	-0.080	-0.053
efitf	0.339***	-0.079*	-0.083*	-0.155***	-0.154***	-0.124**	-0.060	-0.137**	-0.219***
efiif	-0.017*	-0.085***	-0.085***	-0.060***	-0.059***	-0.089***	-0.093***	-0.033**	-0.022**
efiff	-0.004	0.089***	0.091***	0.162***	0.158***	0.094***	0.075***	0.113***	0.120***
lngdppc	0.151***	0.036***	0.036***	0.044***	0.044***	0.098***	0.100***	0.051***	0.049***
R-squared	0.603	0.380	0.380	0.316	0.319	0.302	0.312	0.280	0.316

Note: *** denote 1% significance level, ** denote 5% significance level, * denote 10% significance level. 1 = model without exogenous and endogenous shocks, 2 = model with exogenous and endogenous shocks. Z-statistics are in parentheses.

S5 Table: List of countries – Sampled Countries

List of countries			
Angola	Ghana	Pakistan	Democratic Republic of Congo
Armenia	Guatemala	Panama	Dominican Republic
Azerbaijan	Honduras	Paraguay	Ecuador
Bangladesh	Hungary	Peru	El Salvador
Belize	India	Philippines	Estonia
Bolivia	Indonesia	Poland	Georgia
Bosnia and Herzegovina	Iran	Romania	Mexico
Botswana	Jamaica	Russia	Mongolia
Brazil	Kazakhstan	Serbia	Morocco
Bulgaria	Kenya	Slovakia	Namibia
Burundi	Kyrgyzstan	Slovenia	Nepal
Chile	Laos	South Africa	Nigeria
China	Latvia	Sri Lanka	Uganda
Colombia	Lithuania	Swaziland	Ukraine
Costa Rica	Macedonia	Tanzania	Uruguay
Cote d'Ivoire	Malawi	Thailand	Venezuela
Croatia	Malaysia	Tunisia	Vietnam
Czech Republic	Mauritius	Turkey	Zambia

S6 Table: Cross-sectional dependence test

Cross-sectional dependence tests			
FDIX	149.70***	efigs	151.06***
FIIX	150.03***	efifh	147.46***
FMIX	143.24***	efibf	151.61***
FIDIX	149.21***	efilf	151.61***
FIAIX	147.68***	efimf	151.63***
FIEIX	149.59***	efitf	151.65***
FMDIX	144.61***	efiif	147.70***
FMAIX	109.41***	efiff	151.60***
FMEIX	105.22***	lngdppc	151.67***
efio	151.66***	lnpopg	14.460***
efipr	151.105***	lnGCF	142.84***
efije	151.67***	exogshock	151.41***
efigi	151.17***	endoshock	151.67***
efitb	151.66***		

S1 Appendix: Empirical Method

Linear dynamic panel data GMM-IV

The linear dynamic panel data GMM-IV method estimates panel data on the following equation:

$$y = X\,b + u + e$$

Which is referred to as the untransformed model, also known as, model (level). In the model, y represents the vector of dependent variable's observations, b denotes the coefficients of the regressors, X denotes matrix of independent variables, u represents the group-specific error component, and e is the idiosyncratic error term. Alternatively, a model transformation D orthogonal to u (i.e. $D\,u$ = 0) can be used to get rid of the group-specific error component:

$$D\,y = D\,X\,b + D\,e$$

Model(difference) computes a first-difference transformation from Matrix D, while model(fodev) computes forward-orthogonal deviations, and model(mdev) computes deviations from within-group means, model (mean). The untransformed model, as well as one or more of the transformed models, can have instrumental variables specified [1]. However, it must be remembered that the estimation is not done independently for each model. In fact, the linear dynamic panel data GMM-IV internally transforms the instruments Z for the transformed models into instruments for the untransformed model instead of transforming the estimation equation. On a technical level, this is accomplished by creating the instruments $D'\,Z$ for the original model, where D' is the transpose of the aforementioned transformation matrix.

Panel corrected standard errors and Panel generalised least squares

The panel corrected standard errors method can be used to fit linear cross-sectional time-series models in place of the feasible generalised least squares method in situations in which it is not assumed that the disturbances are independent and are distributed in an identical manner. Alternately, it can be assumed that the disturbances are heteroskedastic across panels, or that they are heteroskedastic and concurrently correlated across panels. In addition, it is possible to hypothesise that the disturbances are autocorrelated within each panel, with the autocorrelation parameter being either the same for all panels or different for each panel. The model can be written as:

$$y_{it} = \boldsymbol{X}_{it}\beta + \epsilon_{it}$$

In the equation, ϵ_{it} is a disturbance that may be autocorrelated along t or contemporaneously correlated across i where i = 1,..., m is the number of units (or panels), t = 1,..., T_i. Where T_i is the number of periods in panel i.

In a panel by panel formular, the model can be simplified as:

$$\begin{bmatrix} \boldsymbol{y}_1 \\ \boldsymbol{y}_2 \\ \vdots \\ \boldsymbol{y}_m \end{bmatrix} = \begin{bmatrix} \boldsymbol{X}_1 \\ \boldsymbol{X}_2 \\ \vdots \\ \boldsymbol{X}_m \end{bmatrix} \beta + \begin{bmatrix} \epsilon_1 \\ \epsilon_2 \\ \vdots \\ \epsilon_m \end{bmatrix}$$

The assumed value of the disturbance covariance matrix in our model, which has heteroskedastic disturbances, contemporaneous correlation, but no autocorrelation, is given as:

$$E[\epsilon\acute{\epsilon}] = \sum_{m\times m} \otimes \mathbf{I}_{T\boldsymbol{i}\times T\boldsymbol{i}}$$

where **I** is an identity matrix and $\sum$ is the panel-by-panel covariance matrix. For this family of models, panel corrected standard errors and generalised least square employ two different

estimation schemes. When autocorrelation is specified, panel corrected standard errors generates Prais-Winsten estimates instead of OLS estimates of the parameters. The estimates of the parameters are dependent on the estimates of the autocorrelation parameter(s) if autocorrelation is specified. When the covariance structure of the disturbances is assumed, the estimation of the variance-covariance matrix of the parameters is asymptotically effective; for more information, see Kmenta [2].

To get complete FGLS parameter and variance-covariance estimates, the panel generalised least squares method could be used. In the spirit of Davidson and MacKinnon [3], Kmenta [2], and Greene [4], estimates of the GLS are conditioned on the disturbance's covariance matrix as well as the autocorrelation parameters. Both panels corrected standard errors and generalised least squares methods are highly consistent and reliable, so far as the conditional mean $\boldsymbol{X}_{it}\beta$ is rightly specified. Moreover, if the assumed covariance structure is appropriate, FGLS estimates computed by panel generalised least square method are more robust and efficient [5].

References

1. Kripfganz S, Schwarz C. Estimation of linear dynamic panel data models with time-invariant regressors. Journal of Applied Econometrics. 2019 Jun;34(4):526-46.
2. Kmenta J, Klein LR. Elements of econometrics. New York: Macmillan; 1971 Dec.
3. Davidson R, MacKinnon JG. Estimation and inference in econometrics. New York: Oxford; 1993.
4. Greene WH. Econometric analysis, 71e. Stern School of Business, New York University. 2012.
5. Beck N, Katz JN. What to do (and not to do) with time-series cross-section data. American political science review. 1995 Sep;89(3):634-47.
