# Supplementary material for: Economic freedom, inclusive growth, and financial development: A heterogeneous panel analysis of developing countries
Source: PLoS One. 2023 Jul 11;18(7):e0288346. doi: 10.1371/journal.pone.0288346 (PMC10335674; doi:10.1371/journal.pone.0288346)
Supplement: Attachment — Submitted filename: REBUTTAL LETTER PLOS ONE.pdf [file pone.0288346.s002.pdf]

pavsackey@gmail.com

9th January 2022

THE EDITOR,

PLOS ONE

Dear Editor,

REBUTTAL LETTER

We write to provide information on changes relative to our recent submission. Below are issues raised in the previous rejected manuscript which have been addressed:

1. "The introduction needs to clearly state at the beginning what exactly is your contribution. At the end of the introduction clearly state what exactly is the gap in the literature and how you intend to address it. Just saying that you are using a set of indices is not good enough. Why is it a better option? What more do you add by using these indices over other variables chosen in past studies?"

   Response
   We have clearly stated our contribution to the literature and have highlighted on the importance of using the set of indices used in the study. Also, we have outlined some related and relevant literature to set out the gap in the literature in which I have filled.

2. "The model should clearly label what the parameters of your estimated equation mean? What do you expect the sign and magnitude to be? What is the long run relationship? Define it."

   Response:
   We highlighted on the parameters in the empirical model and labelled them accordingly. In the long run relationship, we expect economic freedom and inclusive to strongly contribute positively to financial development.

3. "In the empirics you state that the variables are stationary - there is "no unit root". In that case how do you estimate cointegration? Cointegration by definition is a linear combination of I(1) variables. Also, if you reject the unit root null in levels, why test unit roots in differences?"

   Response:
   We have rectified the issues with unit root and cointegration tests. We are most grateful to the reviewer for drawing our attention to this important issue.

4. “The discussion of the results lacks focus and requires sharper interpretation. The overall style of writing needs to be improved.”

   Response:

   We have improved the interpretation of results as well as the discussion. Also, the overall style of writing has been improved.

Yours sincerely,

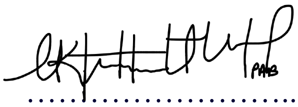

……………………………..

Prince Asare Vitenu-Sackey

Corresponding Author
