# Supplementary material for: Economic freedom, inclusive growth, and financial development: A heterogeneous panel analysis of developing countries
Source: PLoS One. 2023 Jul 11;18(7):e0288346. doi: 10.1371/journal.pone.0288346 (PMC10335674; doi:10.1371/journal.pone.0288346)
Supplement: Attachment — Submitted filename: Responses to reviewers.docx [file pone.0288346.s003.docx]

[pavsackey@gmail.com](mailto:pavsackey@gmail.com)

5^th^ May 2023

THE EDITOR

PLOS ONE JOURNAL

Dear Editor,

**REBUTTAL LETTER**

Please find below our responses to the various comments highlighted for revision for your perusal. In the table, we have particularly addressed all the concerns raised by the academic editor, reviewer one and two, respectively.

| **REVIEW COMMENTS** | **RESPONSES** |
| --- | --- |
| **ACADEMIC EDITOR** |  |
| 1. Rebuttal letter - A rebuttal letter that responds to each point raised by the academic editor and reviewer(s). You should upload this letter as a separate file labeled 'Response to Reviewers'. | Rebuttal letter has been included |
| 2. A marked-up copy of your manuscript that highlights changes made to the original version. You should upload this as a separate file labeled 'Revised Manuscript with Track Changes' | Revised manuscript with track changes has been included |
| 3. An unmarked version of your revised paper without tracked changes. You should upload this as a separate file labeled 'Manuscript' | The original manuscript has been included |
|  |  |
| **REVIEWER ONE** |  |
| 1. Introduction – 3rd sentence. Reference the sentence. | The sentence has been referenced accordingly. |
| 2. The explanation for table 4 should be placed after table 4. This will indicate a better flow of the paper. | Table 4 has been placed under the heading panel unit root and cross-sectional dependence tests which correspond to the explanation. |
| 3. The paper looks very lengthy. Some tables from the results and discussion section can be submitted as supplementary material. | Tables 8, 9, 10, 11 together with the appendix information have all been included attached supplementary results. |
| 4. What was the justification for selecting these specific 72 countries? Countries included are from different regions, sizes, levels of development among many other differences. | The countries were selected based on the IMF's financial development index, with only those scoring below 0.5 out of 1 being considered. |
| 5. How do the findings of your study set out for future research in this area? | Our findings set out the following future research directions:   1. Identify the mechanisms through which tax burden and investment freedom affect financial development. 2. Investigate the impact on other policy variables on financial development. 3. Analyse the effect of financial development on economic growth and possible transmission channels. 4. Consider country-specific variations that could identify the idiosyncratic effect of tax burden, investment freedom and financial development |
|  |  |
| **REVIEWER TWO** |  |
| 1. it would be beneficial if the authors adhere to strict guidelines for in-text referencing, which can be found in the referencing guidelines. | In-text references have been rectified. |
| 2. I recommend that the authors revisit the contributions of their study in the conclusion section and establish a connection with the specific theory being used. | A connection between the theoretical position of the study and our findings have been established and highlighted in the conclusion. |
| 3. since there is a limited amount of literature published in PLOS ONE as relative to the present research, I kindly request that the authors use relevant papers as a guide for their study. | 1. Ding G, Vitenu-Sackey PA, Chen W, Shi X, Yan J, Yuan S. The role of foreign capital and economic freedom in sustainable food production: Evidence from DLD countries. Plos one. 2021 Jul 26;16(7):e0255186 2. Huang J, Ulanowicz RE. Ecological network analysis for economic systems: growth and development and implications for sustainable development. PloS one. 2014 Jun 30;9(6):e100923. 3. Singhal A, Sahu S, Chattopadhyay S, Mukherjee A, Bhanja SN. Using night time lights to find regional inequality in India and its relationship with economic development. PloS one. 2020 Nov 16;15(11):e0241907. |
